# Supplementary material for: Cyclic AMP Regulates Key Features of Macrophages via PKA: Recruitment, Reprogramming and Efferocytosis
Source: Cells. 2020 Jan 6;9(1):128. doi: 10.3390/cells9010128 (PMC7017228; doi:10.3390/cells9010128)
Supplement: Supplementary file 1 [file cells-09-00128-s001.pdf]

Supplementary Materials

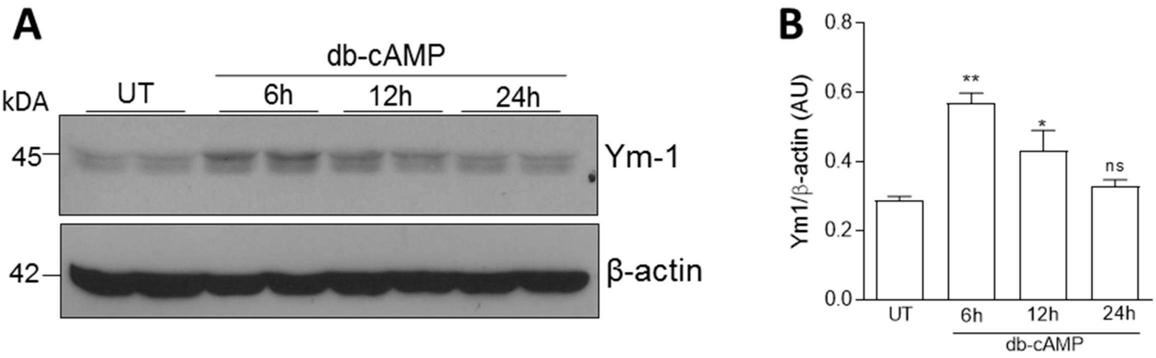

Figure S1. Effects of db-cAMP on Ym-1 expression. BMDMs were treated with db-cAMP (100µM) for 6, 12, or 24 h, and cell lysates were subjected to Western blot analysis to assess Ym-1, a marker of M2 macrophage (A) β-actin was used as a loading control. Densitometry analyses are shown (A). Results are expressed as ratio and are shown as the means ± SEM * $p < 0.05$, ** $p < 0.01$, when comparing with untreated (UT) BMDMs.

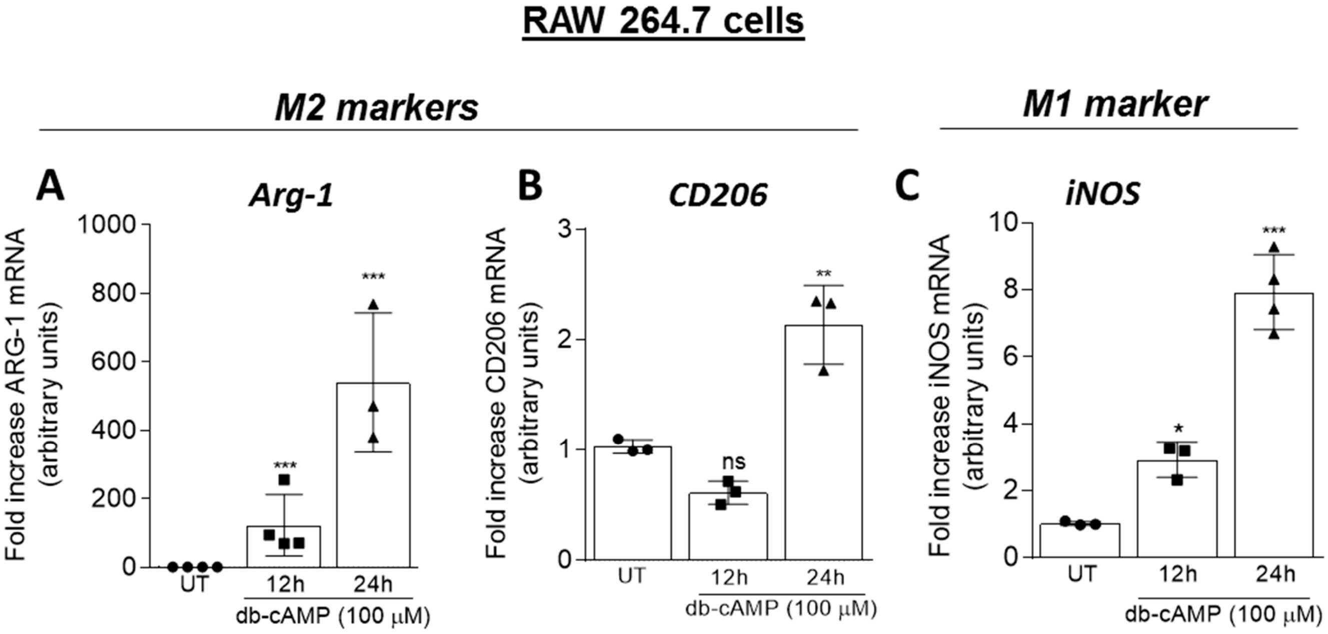

Figure S2. Effect of db-cAMP on polarization of RAW 264.7. RAW 264.7 cells were serum-deprived overnight and treated with db-cAMP (100µM) for 12 and 24 hours and analyzed by qPCR for the expression of the M2 markers arginase-1 (A) and CD206 (B) and the M1 marker iNOS (C). Results are expressed as fold increase and are shown as the means ± SEM * $p < 0.05$, ** $p < 0.01$, *** $p < 0.001$ when comparing with untreated (UT) cells.

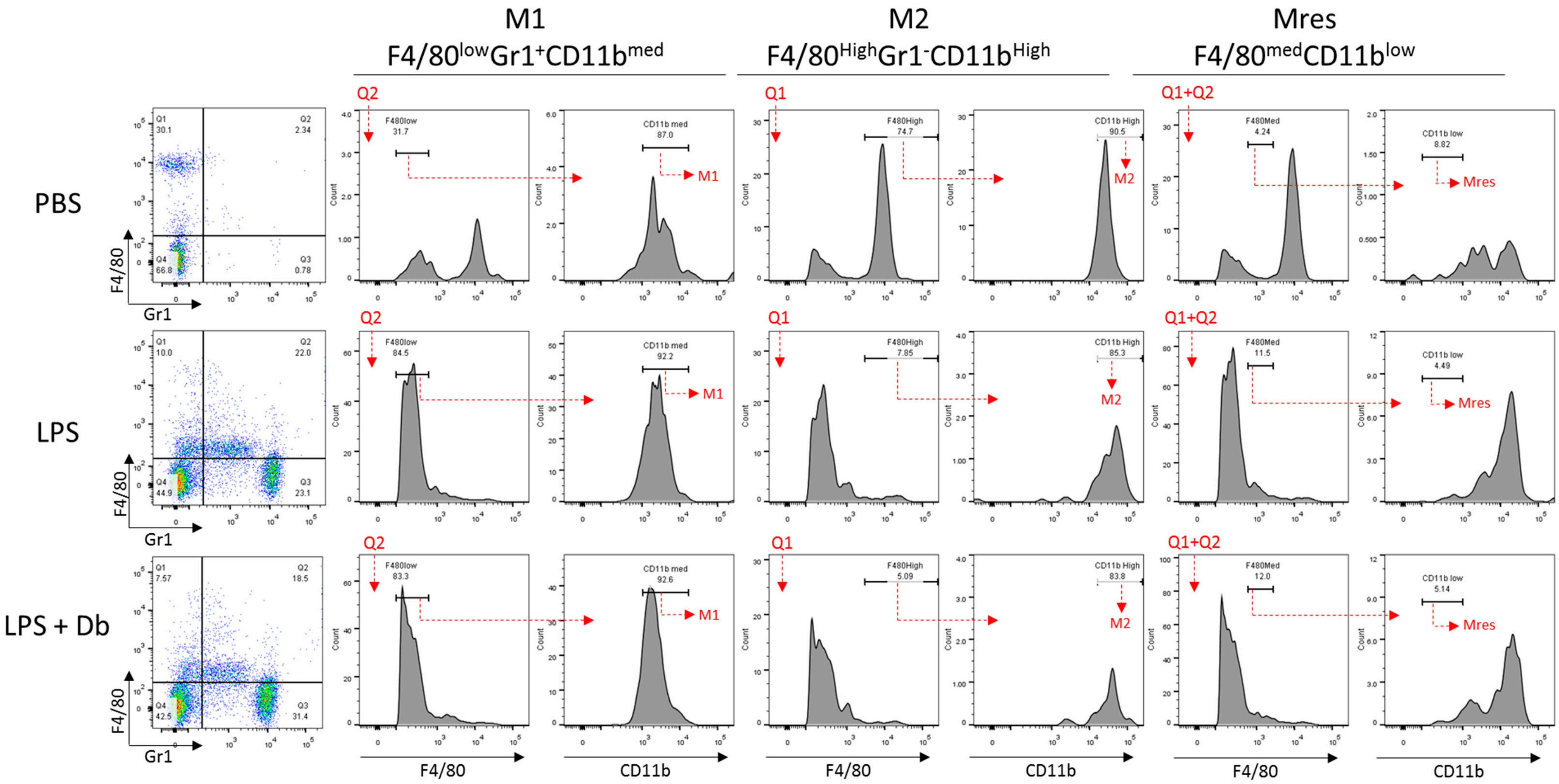

Figure S3. Gating scheme and representative dot plots and histograms showing the effect of db-cAMP on macrophage polarization of LPS-inflamed mice. BALB/c mice were injected with LPS (250 ng/cavity, i.pl.) or PBS, and 8 h later received an injection of db-cAMP (4 mg/kg, i.pl.). Cells present in the pleural cavity were harvested 30 hours after LPS challenge or PBS injection. Cells were immunostained for Gr1, F4/80, and CD11b, and analyzed by FACS. Leukocytes were initially gated based on forward and side scatters. Then,

singlets were defined using forward scatter height and area (not shown). The next step was to identify macrophage populations based on F4/80 and Gr1 staining (dot plots on the left). F4/80$^+$Gr1$^-$ (first quadrant, Q1), F4/80$^+$Gr1$^+$ (Q2) and total F4/80$^+$ (Q1+Q2) populations were further gated according to the level of expression (low, medium or high) of F4/80 and CD11b surface markers (histograms). For M1 macrophages, cells from the second quadrant (Q2) were analyzed for the expression of F4/80. Next, F4/80^{low} cells were analyzed for CD11b expression, of which CD11b^{med} were identified. Similarly, cells from the first quadrant (Q1) were analyzed for the expression of F4/80. F4/80^{high} cells were then analyzed for CD11b expression, of which CD11b^{high} cells were identified. Finally, cells from the first and second quadrants (Q1+Q2) were analyzed for the expression of F4/80. Next, the F4/80^{med} population was analyzed for CD11b expression, of which CD11b^{low}were identified. The frequency of M1 [F4/80^{low} Gr1$^+$ CD11b^{med}], M2 [F4/80^{high} Gr1$^-$ CD11b^{high}], and Mres [F4/80^{med} CD11b^{low}] macrophages in the whole population of single cells was used to calculate the number of each macrophage phenotype present in the pleural exudate (frequencies of M1, M2 or Mres in the population of single cells x total number of cells on the pleural cavity), as shown in Figure 5.
